# Cost-Effectiveness of Internet-Delivered Emotion Regulation Therapy for Adolescents With Nonsuicidal Self-Injury: Within-Trial Analysis of a Randomized Controlled Trial

**DOI:** 10.2196/74303

**Published:** 2025-08-27

**Authors:** Johan Bjureberg, Olivia Ojala, Björn Rasmusson, Jessica Malmgren, Clara Hellner, Filipa Sampaio, Oskar Flygare

**Affiliations:** 1 Centre for Psychiatry Research, Department of Clinical Neuroscience, Karolinska Institutet, & Stockholm Health Care Services, Norra Stationsgatan 69, Stockholm, 113 64, Sweden, 46 730917112; 2 Department of Public Health and Caring Sciences, Uppsala University, Uppsala, Sweden

**Keywords:** cost-effectiveness, emotion regulation therapy, internet-delivered treatment, nonsuicidal self-injury, self-harm

## Abstract

**Background:**

Nonsuicidal self-injury (NSSI) is common among adolescents and is associated with adverse clinical outcomes, as well as suicidal behavior. Current treatments are resource-intensive and may not be accessible to all adolescents with NSSI. Internet-delivered emotion regulation individual therapy for adolescents (IERITA) with NSSI disorder is a promising treatment option, but its cost-effectiveness is unknown.

**Objective:**

This study aims to evaluate the cost-effectiveness of IERITA for adolescents with NSSI disorder.

**Methods:**

Within-trial cost-effectiveness analysis of a randomized controlled trial at three child and adolescent mental health services in Sweden (n=166). A total of 12 weeks of IERITA plus treatment as usual (TAU) versus TAU only were compared. The primary outcome was the frequency of NSSI at 1-month posttreatment. Secondary outcomes were NSSI remission and quality-adjusted life years (QALYs).

**Results:**

IERITA led to reductions in NSSI frequency, a higher proportion of participants with NSSI remission, and more QALYs at 1-month posttreatment, at additional health care costs of US $3663 (95% CI US $2182-$5002) and societal costs of US $4458 (95% CI US $−577 to $9509). The incremental cost of one additional NSSI remission at 1-month posttreatment was US $18,677, and the incremental cost per QALY gained was US $792,244 for IERITA+TAU relative to TAU. IERITA had an 8% probability of being cost-effective at a societal willingness-to-pay threshold of US $84,000 for one QALY at 1-month posttreatment, which increased to 18% at 3-months posttreatment.

**Conclusions:**

IERITA delivered adjunctive to TAU led to improvements in NSSI frequency, remission, and QALYs, at additional costs compared to TAU only. This study provides an estimate of the additional cost of delivering IERITA; however, future studies should include longer follow-up periods to better assess the magnitude of the effects on QALYs and societal costs.

## Introduction

Nonsuicidal self-injury (NSSI), the damaging of one’s own tissue without suicidal intent, is a strong risk factor for future suicidal behavior and adverse outcomes [1,2]. As knowledge about NSSI has improved, NSSI disorder (NSSID) was included as a condition for further study in the 5th edition of the Diagnostic and Statistical Manual of Mental Disorders [3]. The proposed criteria for NSSID include: (1) engagement in NSSI on at least 5 days in the past year (Criterion A); (2) expectation that NSSI will solve an interpersonal problem, provide relief from unpleasant thoughts or emotions, or induce a positive state (Criterion B); (3) experiencing one or more of the following: interpersonal problems or negative emotions prior to NSSI, difficulty managing preoccupation with NSSI, or frequent thoughts about NSSI (Criterion C); (4) NSSI is not socially sanctioned or to minor self-injurious behaviors (Criterion D); (5) clinically significant distress or interference in functioning due to NSSI (Criterion E); and (6) NSSI is not solely due to psychosis, delirium, substance use or withdrawal, or another psychiatric disorder or medical condition (Criterion F). While as many as 35.6% of a community sample of adolescents have been shown to engage in past-year NSSI, 6.7% met the suggested Diagnostic and Statistical Manual of Mental Disorders, 5th edition criteria [4,5]. By age 19, the likelihood of favorable outcomes—defined as the absence of mental health or substance use problems and engagement in education or employment—is reduced by half among individuals who experienced NSSI or self-injury with suicidal intent at age 16 compared to peers without such histories [6].

In addition to the clinical implications, NSSI is associated with economic strain for the afflicted individual and their close relatives, as well as costs for the health care sector and society. For example, a recent Danish registry-based study estimated that the mean hospital cost per presentation due to self-harm was US $7029 [7]. A large proportion of that cost is due to inpatient psychiatric care, which is common after NSSI in both adults and adolescents [8,9]. Extended periods of inpatient psychiatric care are disruptive for the individual, who may experience stigma and loss of social support [10]. NSSI in youth is associated with school absenteeism and academic underachievement, which pose short- and long-term challenges [11]. Further, parents of youth with self-harm report having to take time off work to care for their child, which may extend to sick leave and unemployment [12]. To mitigate the immediate consequences and prevent negative long-term outcomes, it is crucial to offer effective treatment for NSSI promptly, considering both the direct and indirect costs involved.

When deciding among several treatment options, decision makers in health care are weighing treatment effects against costs. In the case of NSSI in youth, the only well-established treatment with two independent trials to support its efficacy is dialectical behavior therapy for adolescents (DBT-A [13]). While effective in reducing NSSI, this lengthy and resource-intensive treatment remains inaccessible for most adolescents with NSSI [14-16]. In a randomized controlled trial of DBT-A in Norway, for example, therapists (psychologists or psychiatrists) received 80 hours of training and 12 months of supervised practice and were assessed as being adherent to the treatment protocol before providing treatment to patients [14]. In the same trial, delivering DBT-A over 19 weeks incurred additional costs of €15,850 (US $15,052) per patient [17]. Despite the relatively large costs associated with DBT-A in the Norwegian trial, it was cost-effective at 1-year follow-up compared to enhanced usual care, due to reducing self-harm and lowering inpatient treatment costs [17]. However, given the level of resources and costs associated with DBT-A, previous trials have offered DBT-A to adolescents also presenting with symptoms of borderline personality disorder and high risk of suicide [14-16]. To address the need for a brief and scalable intervention that can be implemented early in the trajectory of NSSI, our group has developed a digital internet-delivered emotion regulation individual therapy for adolescents (IERITA) targeting those presenting with NSSI. IERITA has been shown to be acceptable and effective in reducing NSSI in multiple pilots [18-20] and a randomized controlled trial enrolling 166 adolescents and their parents [21]. However, a formal cost-effectiveness analysis is needed before the treatment can be implemented alongside other treatment options. The aim of this study was to conduct a health economic evaluation of IERITA, using data from a recent randomized controlled trial [21].

## Methods

### Trial Design

This study reports within-trial cost-effectiveness analyses from a 3-site randomized clinical trial of IERITA plus treatment as usual (TAU) versus TAU only for adolescents with NSSID and their parents (n=166) [21]. After an initial telephone screening, participants completed self-reported assessments and a face-to-face assessment at one of the participating clinics. Eligible participants were then randomly allocated to IERITA plus TAU or TAU only and started the respective treatments, after which 1-month posttreatment (primary end point) and 3-months posttreatment assessments were completed. For a full description of the study procedures, please see the initial report of the trial [21]. The results are reported in accordance with the Consolidated Health Economic Evaluation Reporting Standards 2022 (CHEERS 2022) statement [22].

### Ethical Considerations

The trial was approved by the Stockholm Regional Ethical Review Board (2017/1807‐31). All participants provided informed consent, with older participants providing written consent and younger participants providing verbal consent along with parental written consent. Participants could withdraw from the study at any given time without any negative consequences. Adolescents in both the IERITA plus TAU and TAU only conditions were entitled to receive reimbursement if they completed certain self-report measures and participated in the clinician-administered interviews (up to approximately US $50 in the IERITA plus TAU condition and US $110 in the TAU only condition).

### Participants

Eligible participants were adolescents aged between 13 and 17 years who fulfilled diagnostic criteria for NSSID, had experienced one or more NSSI episodes during the past month, and had one guardian who could participate in the parent program. Exclusion criteria were an immediate suicide risk, diagnosis of psychotic or bipolar I disorder or past-month substance use disorder, another primary psychiatric disorder requiring immediate treatment (eg, severe anorexia nervosa), insufficient understanding of the Swedish language, life circumstances that could prevent treatment participation or that require immediate intervention, and a Children’s Global Assessment Scale score below 40. Detailed information on the study sample is presented in Table 1.

**Table 1.** Sociodemographic and clinical characteristics of the sample.

	IERITA^a^ plus TAU (n=84)	TAU^b^ only (n=82)	Total (n=166)
Sex, n (%)			
Female	77 (92)	77 (94)	154 (92.8)
Male	5 (6)	2 (2)	7 (4.2)
Nonbinary	2 (2)	3 (4)	5 (3)
Age (years), mean (SD)	15.04 (1.31)	15.02 (1.19)	15.03 (1.25)
Age NSSI^c^ onset (years), mean (SD)	12.7 (1.27)	12.51 (1.57)	12.61 (1.42)
Comorbidity, n (%)			
Major depressive disorder	49 (58)	48 (58.5)	97 (58.4)
Social anxiety disorder	24 (29)	23 (28)	47 (28.3)
Panic disorder or agoraphobia	17 (20)	11 (13.4)	28 (16.9)
Specific phobia disorder	14 (17)	13 (16)	27 (16)
Generalized anxiety disorder	12 (14)	9 (11)	21 (12.6)
ADHD^d^	14 (17)	15 (18)	29 (18)
Autism spectrum disorder	4 (5)	3 (4)	7 (4)
OCD^e^ or BDD^f^	3 (4)	7 (9)	10 (6)
Eating disorder	6 (7)	1 (1)	7 (4)
Oppositional defiant disorder	3 (4)	2 (2)	5 (3)
Any previous suicide attempt, n (%)	13 (16)	12 (15)	25 (15)
Previous counseling, n (%)	54 (64)	53 (65)	107 (64)
Time in previous counseling (months), mean (SD)	10.6 (13.8)	10.2 (14.8)	10.4 (14.3)
Ever received inpatient care, n (%)	2 (2)	2 (2)	4 (2)
Ongoing counseling at inclusion, n (%)	61 (73)	55 (67)	116 (67)
Ongoing psychopharmacological medication at inclusion, n (%)	31 (37)	25 (31)	56 (34)
Baseline NSSI frequency, mean (95% CI)	2.82 (2.48-3.16)	2.77 (2.42-3.12)	N/A^g^
Baseline CHU-9D^h^ utility, mean (95% CI)	0.635 (0.627-0.644)	0.630 (0.621-0.638)	N/A
Baseline total health care costs (US $), mean (SD)	4492 (1741)	4297 (1739)	N/A
Baseline total societal costs (US $), mean (SD)	24212 (8322)	21239 (8322)	N/A

a IERITA: internet-delivered emotion regulation individual therapy for adolescents.

b TAU: treatment as usual.

c NSSI: nonsuicidal self-injury.

d ADHD: attention-deficit hyperactivity disorder.

e OCD: obsessive-compulsive disorder.

f BDD: body dysmorphic disorder.

g Not applicable.

h CHU-9D: Child Health Utility 9 Dimensions.

### Interventions

The therapist-guided IERITA treatment is an acceptance-based behavioral treatment delivered through a web-based platform [18,20,23]. The treatment includes 11 digital modules for the adolescent participant and 6 modules for the parents, delivered over the course of 12 weeks with asynchronous support from a dedicated therapist. The treatment targets the role of emotion dysregulation in maintaining NSSI, and the treatment aims to teach participants more adaptive ways of responding to their emotions. The treatment also includes modules on impulse control training, self-validation, valued direction, and relapse prevention. IERITA was delivered adjunctive to TAU. Adolescents receiving IERITA completed a mean (SD) of 9.6 modules out of 11, and five participants dropped out of the treatment [21].

TAU consisted of continued care within regular child and adolescent mental health services or referral to such services if deemed necessary after the baseline assessment. TAU was delivered in both groups through standard child and adolescent mental health service care by community clinicians (not part of the research team), usually consisting of supportive therapy sessions biweekly. TAU was enhanced by weekly self-rated assessments and follow-ups from research clinicians as needed. Communication between community therapists and IERITA therapists only occurred as a safety measure when deterioration in mental health was detected.

### Resource Use and Costs

Intervention costs were estimated by multiplying the therapist’s time spent on treatment by the average hourly cost of a psychologist in outpatient child and adolescent psychiatric services. The therapist’s time spent on the platform was automatically recorded, and therapists manually logged time spent on phone calls following guidelines developed by the study team. Other resources used by participants were collected using the parent-rated version of the Trimbos-iMTA questionnaire for costs associated with psychiatric illness (TIC-P) [24] at pretreatment, 1-month posttreatment (16 wk after pretreatment), and at 3-months posttreatment (24 wk after pretreatment) with a 12-week recall period (adjusted to 8 wk at 3 mo posttreatment to cover the period since 1 mo posttreatment). The TIC-P included health care visits, prescription medication, and supplements, as well as resource use beyond health care, including social support and assistance, school absenteeism and presenteeism for children, and absenteeism from both paid and unpaid work for parents (also including time spent engaging with treatment content and supporting their child). Costs were estimated by multiplying resource use frequencies by unit costs and adding them to the intervention costs. Health care costs were estimated by multiplying the number of visits by the unit cost of a visit using tariffs from official listings for the Swedish health care system (South-East region); costs for medications were estimated by using individual product prices from The Dental and Pharmaceutical Benefits Agency of Sweden [25], and costs for supplements were estimated using individual product prices from Swedish pharmacies. Costs for social support and assistance were estimated using tariffs for support family placement and personal assistant from Sweden’s municipalities and regions, and study help costs from companies who provide the service at an hourly rate (from TIC-P: “how many hours of [study help or support family placement or personal assistant] have you used in the past 3 months?”). Support from family and friends was costed using the average posttax wage per hour in Sweden [26,27]. Parental productivity losses related to absenteeism from paid work were estimated using the human capital approach and calculated as the average salary in Sweden plus social fees multiplied by the number of days absent from work [27,28]. Parental productivity losses related to absenteeism from unpaid work were estimated using the average posttax wage per hour in Sweden multiplied by the number of hours reported being absent from unpaid work [27]. Productivity losses for children related to absenteeism from school were calculated as the average annual cost per pupil and year divided by the number of school days per year and multiplied by the number of days reported being absent from school. Productivity losses for children related to presenteeism at school were estimated in a similar manner as school absenteeism–related costs but multiplied by the number of days with lower performance at school and by the percentage loss in performance (from TIC-P: “on days not feeling well, how much is your child able to participate in school compared to days feeling well? Rate on a scale of 0%‐100%”). Additional details are provided in Table S1 in Multimedia Appendix 1.

The health care perspective included costs related to health care visits, prescribed medication, and supplements not requiring a prescription, in addition to the intervention costs. The societal perspective additionally included costs for social support and assistance, productivity losses related to school absenteeism and presenteeism for children, and productivity losses related to absenteeism from paid and unpaid work for parents.

Total costs over the trial period were calculated for each randomized participant and aggregated over time. Resources were costed in 2018 Swedish Krona, uprated to 2022 using Consumer Price Index from Statistics Sweden [29], and converted to 2022 US $ using purchasing power parities for gross domestic product [30]. As all data were considered within 1 year, no discounting was applied.

### Health Outcomes

The primary clinical measure, change in NSSI frequency from pretreatment to posttreatment, was assessed using the Deliberate Self-Harm Inventory-Youth version (DSHI-Y) [31]. The DSHI-Y is an adapted and shortened version of the DSHI [32]. The DSHI has demonstrated high internal consistency (α=0.82) and test-retest reliability over a period of 2 to 4 weeks [32]. The DSHI-Y consists of 6 items that encapsulate the most common methods of NSSI in youth: cutting, burning, severe scratching, self-biting, banging (banging head or other body parts against an object), and self-punching, resulting in injury severe enough for tissue damage (eg, scarring or bruises) to occur. Clinician ratings of DSHI-Y, assessing NSSI frequency in the past month (ie, NSSI remission), were administered pretreatment, 1-month posttreatment, and 3-months posttreatment by masked assessors. The proportion of individuals with no NSSI in the past month was calculated based on the clinician ratings at posttreatment. In addition, self-reported DSHI-Y was administered pretreatment, once every week in treatment, and 4 weeks posttreatment.

Participants’ health-related quality of life was measured using the Kid-Screen 10. The Kid-Screen 10 is a short self-rated assessment of health-related quality of life that was administered at pretreatment, 1-month posttreatment, and 3-months posttreatment. The questionnaire measures the young person’s subjective health and quality of life in the domains of physical and mental well-being, independence, school environment, and relationship to parents and friends [33]. Kid-Screen 10 has adequate internal consistency (α=0.81) [34]. Since the Kid-Screen 10 does not make it possible to calculate quality-adjusted life years (QALYs) needed for cost-utility analysis, participant responses on the Kid-Screen 10 were mapped onto Child Health Utility 9D (CHU9D) utility scores in order to estimate QALYs, using an algorithm with a mean absolute error of 0.0946 [35]. The CHU9D questionnaire measures health-related quality of life on 9 dimensions (eg, worried, sad, schoolwork, and activities) in children [36], and has a validated algorithm for calculating QALYs [37]. Total QALYs during the trial period were calculated by using the area under the curve method, assuming a constant rate of change between assessment points [38].

### Statistical Analyses

The cost-effectiveness analyses used the following outcomes: NSSI frequency and proportion of participants who experienced NSSI remission in the past month, assessed by the masked clinician-assessed DSHI-Y, and weekly NSSI frequency during treatment, assessed by the DSHI-Y. The cost-utility analyses used QALYs from CHU9D as the outcome. All analyses were conducted from the health care sector and the societal perspectives.

Total costs over the trial period were aggregated by trial arm and presented as descriptive statistics. No extrapolation beyond the recall period of 12 weeks was used, and the costs at 1-month posttreatment, therefore, included all costs from week 4 in treatment up until the 1-month posttreatment assessment point. The statistical analyses were conducted on an intention-to-treat basis, and missing data were imputed using multiple imputation by chained equations and predictive mean matching, under the assumption that data were missing at random [39,40]. The statistical models did not include the study site as a covariate or clustering variable. Sensitivity analyses using only complete data with no missing data imputation were also performed.

Group differences in NSSI reductions over time were estimated using a zero-inflated negative binomial generalized linear mixed effects regression model, including an interaction term between treatment condition and time, as well as random intercept and slope. The resulting incidence rate ratios (IRRs) were then used as the outcome of interest. This model was also used as the primary outcome analysis in the clinical trial from which data were used [21].

Cost data, NSSI remission, and QALYs were fitted using seemingly unrelated regression models at 1-month posttreatment and 3-months posttreatment, respectively, combined with nonparametric bootstrap estimation (5000 iterations) within each multiply imputed dataset [41,42]. This allowed for robust modeling of skewed distributions in both costs and effects while simultaneously adjusting for baseline imbalances and missing data [43,44]. Between-group differences in costs were analyzed while controlling for baseline costs, and QALYs were analyzed while controlling for baseline CHU9D utilities [45]. The analyses are presented as incremental cost-effectiveness ratios (ICER; the between-group mean difference in costs divided by the between-group mean difference in health outcome) [46] with CIs calculated using the bootstrap percentile method [47], and cost-effectiveness planes (bootstrap estimates of cost differences displayed on the y-axis and estimates of health outcome differences on the x-axis, indicating if the intervention is more or less costly and effective compared to the alternative) [48]. Net monetary benefits at different thresholds of willingness to pay (ie, how much the society is willing to pay for a one-unit difference in the health outcome) were calculated for mean values and are presented on cost-effectiveness acceptability curves (CEACs), which capture decision uncertainty and show the probability of IERITA being cost-effective at different thresholds of willingness to pay for improvement on the health outcomes [46,49]. Willingness to pay depends on the country and the structure of the health care system [50]. There is currently no threshold defined in Sweden, and we used a threshold reported in a review of reimbursement decisions for pharmaceuticals in Sweden [51].

The preregistered data analysis plan and statistical code used in this study can be found on the Open Science Framework [52]. The methods described in this publication differ from the preregistration in that the costs have been uprated to 2022 and that the statistical models for costs and QALYs used seemingly unrelated regression rather than specifying the underlying distributions in generalized linear models, in accordance with guidance on within-trial cost-effectiveness studies [43,44].

## Results

### Completion Rates and Participant Characteristics

**Figure 1.**
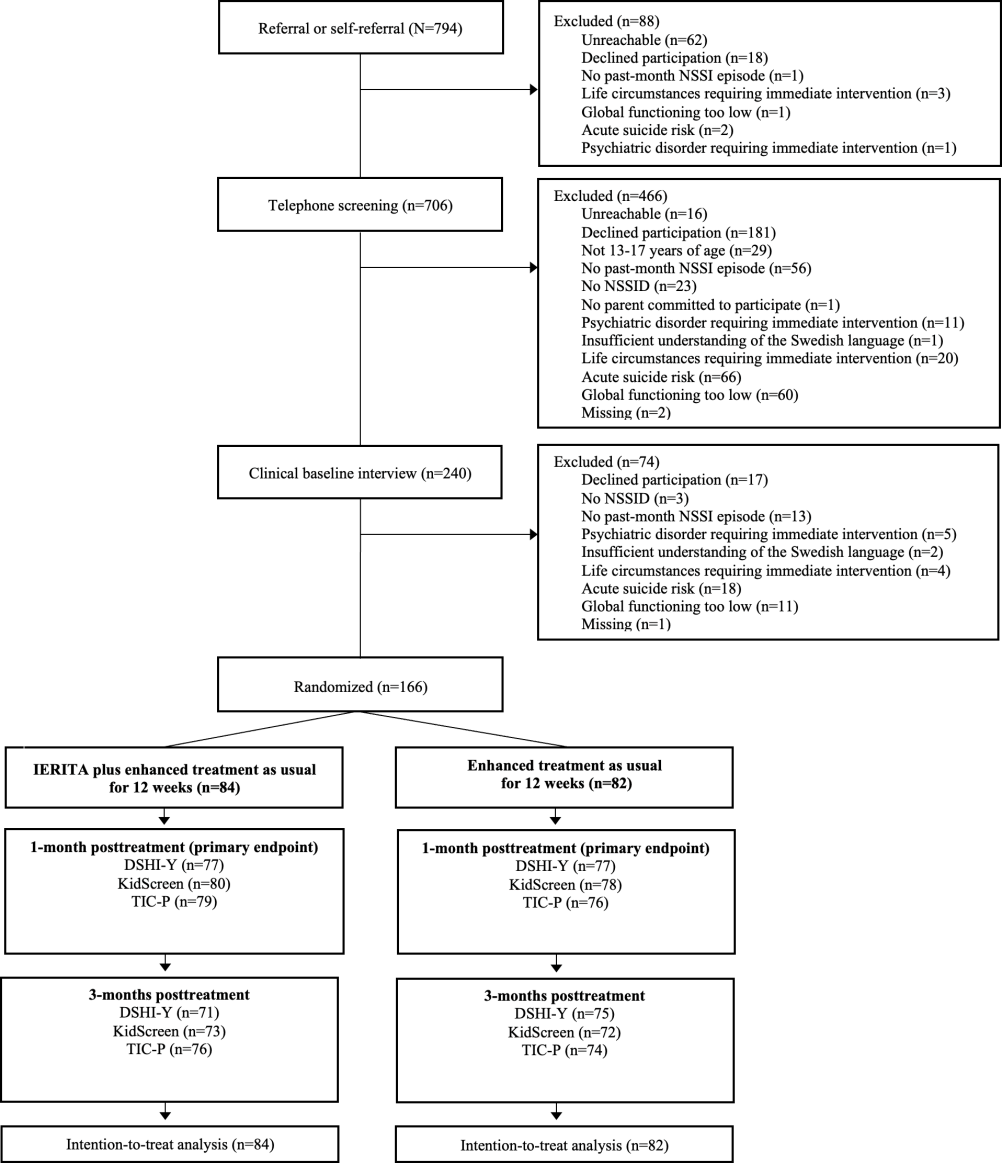
Flow diagram of patient enrollment. DSHI-Y: Masked Assessor-rated Deliberate Self-Harm Inventory-Youth Version; IERITA: internet-delivered emotion regulation individual therapy for adolescents; NSSI: nonsuicidal self-injury; NSSID: nonsuicidal self-injury disorder; TIC-P: Trimbos-iMTA questionnaire for costs associated with psychiatric illness.

All participants completed the baseline assessments on all outcomes. At the 1-month posttreatment assessments, completion rates in the IERITA plus TAU group were 92%‐95% (DSHI-Y: n=77, 92%; KidScreen: n=80, 95%; TIC-P: n=79, 94%), and completion rates in the TAU only group were 93%‐95% (DSHI-Y: n=77, 94%; KidScreen: n=78, 95%; TIC-P: n=76, 93%). The completion rates at 3-months posttreatment were 85%‐90% in the IERITA plus TAU group (DSHI-Y: n=71, 85%; KidScreen: n=73, 87%; TIC-P: n=76, 90%) and 88%‐91% in the TAU only group (DSHI-Y: n=76, 91%; KidScreen: n=72, 88%; TIC-P: n=74, 90%; Figure 1). Data on therapist time spent on treatment were missing for 10 (6%) participants. The majority of participants were female, and most were enrolled in either pharmacological or psychological treatment as baseline (see Table 1 for patient characteristics).

There was no difference in health care sector costs between the two groups at baseline (IERITA plus TAU: US $4492, 95% CI US $4119-$4865; TAU only: US $4297, 95% CI US $3919-$4675; *P*=.47), however, the IERITA plus TAU group had higher societal costs (IERITA plus TAU: US $24,212, 95% CI US $22,430-$25,994; TAU only: US $21,239, 95% CI US $19,436-$23,042; *P*=.02).

There were statistically significant correlations between estimated societal costs and both NSSI remission (*r*=−0.32; *P*<.001) and QALYs (*r*=−0.2; *P*<.001) at 1-month posttreatment, supporting the use of seemingly unrelated regression to model costs and health outcomes simultaneously.

### Costs

At 1-month posttreatment, the cost of providing IERITA adjunctive to TAU was estimated to US $3552 (SE 54.1), and IERITA plus TAU was associated with higher costs compared to TAU only from a health care perspective (US $3663, 95% CI US $2182-$5002) and societal perspective (US $4458, 95% CI US −$577 to $9509). These differences remained similar in magnitude at 3-months posttreatment (US $3440, 95% CI US $1455-$5319 and US $4565, 95% CI US −$2820 to $11,919 for health care and societal costs, respectively). Table 2 provides an overview of estimated costs and health outcomes over the trial period, including bootstrap estimates adjusting for baseline imbalances. Table 3 shows health care resource use in enhanced TAU (eTAU) during the trial period for both groups.

**Table 2.** Estimated costs and health outcomes over the trial period^a^.

Outcome	Estimated mean^b^ (SE)		Mean difference	
	IERITA^c^ plus TAU^d^ (n=84)	TAU only (n=82)	Unadjusted^e^	Adjusted (95% CI)^f^
1-month posttreatment				
Costs (US $)				
Health care costs	7658 (238)	3926 (241)	3733^g^	3663 (2182 to 5002)
Societal costs	23,758 (953)	18,265 (965)	5493^g^	4458 (−577 to 9509)
Health outcomes				
NSSI^h^ frequency	1.29 (95% CI 0.94 to 1.63)	2.21 (95% CI 1.87 to 2.54)	0.92^i^	N/A^j^
NSSI remission	0.61 (0.02)	0.38 (0.02)	0.23^g^	0.23 (0.08 to 0.39)
QALYs^k^	0.205 (0.01)	0.199 (0.01)	0.006^g^	0.005 (0.001 to 0.01)
3-months posttreatment				
Costs (US $)				
Health care costs	9649 (320)	6126 (324)	3524^g^	3440 (1455 to 5319)
Societal costs	34,559 (1402)	28,328 (1419)	6231^l^	4565 (−2820 to 11,919)
Health outcomes				
NSSI frequency	1.17 (95% CI 0.88 to 1.46)	1.68 (95% CI 1.30 to 2.06)	0.51^i^^,^^m^	N/A
NSSI remission	0.58 (0.02)	0.51 (0.02)	0.07^m^	0.06 (−0.10 to 0.21)
QALYs	0.313 (0.002)	0.302 (0.002)	0.010^g^	0.009 (0.001 to 0.017)

a Costs and QALYs are accumulated from baseline until follow-up, NSSI frequency shows estimated values from baseline to follow-up and NSSI remission shows between-group differences at follow-up.

b Estimated means from the multiply imputed datasets.

c IERITA: internet-delivered emotion regulation individual therapy for adolescents.

d TAU: treatment as usual.

e *P* values not adjusted for multiple testing.

f Adjusted mean differences from 5000 bootstrap samples while controlling for baseline costs in the same category (costs) or baseline CHU9D utilities. No adjustment for NSSI remission differences.

g *P*<.001.

h NSSI: nonsuicidal self-injury.

i Incidence rate ratio=0.92 (95% CI 0.88-0.91); *P*<.001. Values indicate the estimated reduction in NSSI frequency of IERITA plus TAU compared to TAU only.

j Not applicable.

k QALY: quality-adjusted life year.

l *P*<.01.

m *P*<.05.

**Table 3.** Characteristics of enhanced TAU^a^.

Characteristic	Participants, n (%)	
	IERITA^b^ plus TAU	TAU only
Baseline to 1-month posttreatment^c^		
Received counseling	62 (78.5)	56 (73.7)
Frequency of counseling		
Every week	11 (17.8)	13 (23.2)
Every second week	18 (29)	20 (35.7)
Once per month	23 (37.1)	14 (25)
Less than once per month	9 (14.5)	9 (16.1)
Never	1 (1.6)	0 (0)
Inpatient care	0 (0)	1 (1.3)
Any ongoing psychopharmacological medication	32 (46)	35 (41)
1-month posttreatment to 3-months posttreatment^d^^,^^e^		
Received counseling	48 (64.9)	52 (70.3)
Frequency of counseling		
Every week	7 (14.6)	12 (23.1)
Every second week	11 (22.9)	13 (25)
Once per month	16 (33.3)	14 (26.9)
Less than once per month	14 (29.2)	13 (25)
Never	0 (0)	0 (0)
Inpatient care	0 (0)	1 (1.4)
Any ongoing psychopharmacological medication	35 (46)	31 (42)

a TAU: treatment as usual.

b IERITA: internet-delivered emotion regulation therapy for adolescents.

c For baseline to 1-month posttreatment, IERITA plus TAU included 79 participants and TAU only included 76 participants.

d For 1-month posttreatment to 3-months posttreatment, both IERITA plus TAU and TAU only included 74 participants.

e Pharmacological results included 76 participants in the IERITA plus TAU group.

### Health Outcomes

There were fewer NSSI episodes on the masked clinician-assessed DSHI-Y at 1-month posttreatment in the IERITA plus TAU group compared to TAU only (IRR 0.38, 95% CI 0.24-0.60; *P*<.001), and IERITA plus TAU also resulted in fewer NSSI episodes at 3-months posttreatment compared to TAU only (IRR 0.57, 95% CI 0.36-0.90; *P*=.02). The estimated proportion of participants with no NSSI at 1-month posttreatment was greater in the IERITA plus TAU group (61%, 95% CI 56%-66%) compared to TAU only (38%, 95% CI 34%-43%), a difference that was statistically significant (odds ratio=2.5; SE=0.4; *P*<.001). At 3-months posttreatment, the difference was attenuated but remained statistically significant (IERITA plus TAU: 58%, 95% CI 53%-63%; TAU only: 51%, 95% CI 46%-56%; odds ratio=1.33; SE=0.19; *P*=.04). Results on the self-rated DSHI-Y were similar, with larger improvements in the IERITA plus TAU group compared to TAU only (Multimedia Appendix 1). The IERITA plus TAU group generated more QALYs compared to TAU only between pretreatment and 1-month posttreatment (*b*=0.006, 95% CI 0.002-0.010; SE=0.002; *P*<.001), and between pretreatment and 3-months posttreatment (*b*=0.01, 95% CI 0.005-0.016; SE=0.003; *P*<.001).

### Cost-Effectiveness and Cost-Utility

#### The Societal Perspective

**Figure 2.**
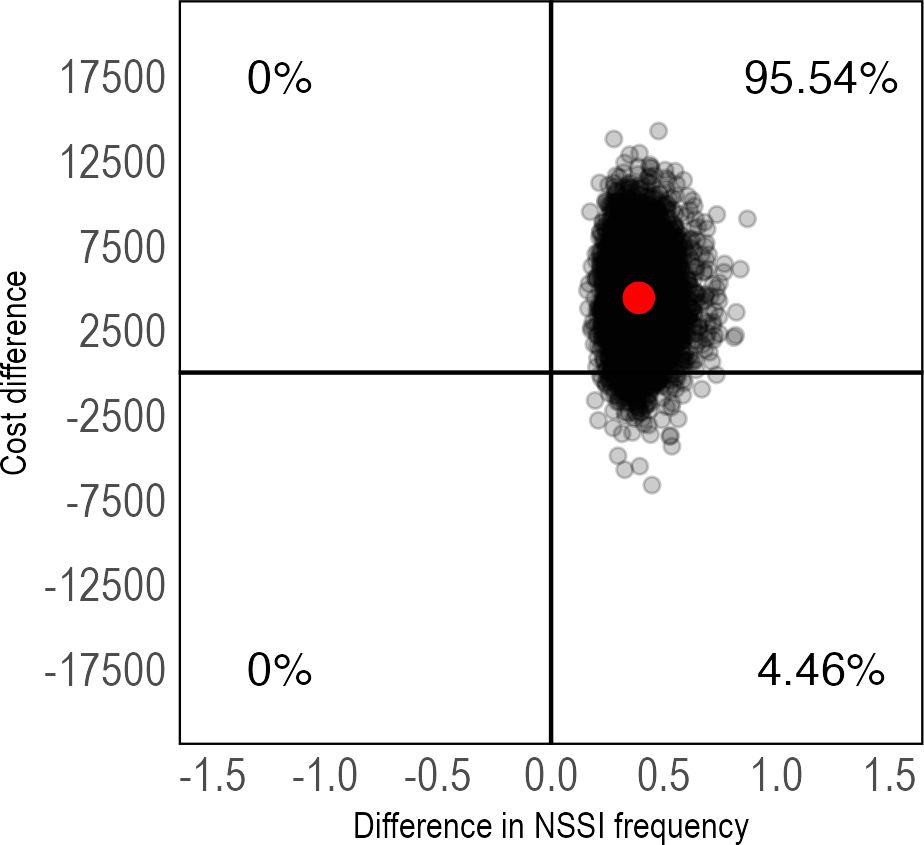
Pretreatment to 1-month posttreatment cost-effectiveness plane of IERITA plus TAU compared to TAU only, NSSI frequency. The x-axis displays between-group differences in outcomes, with positive values indicating more improvement in the IERITA plus TAU group compared to TAU only. Differences in costs, shown on the y-axis, are from a societal perspective, with positive values indicating increased costs in the IERITA plus TAU group compared to TAU only. IERITA: internet-delivered emotion regulation individual therapy for adolescents; NSSI: nonsuicidal self-injury; TAU: treatment as usual.

**Figure 3.**
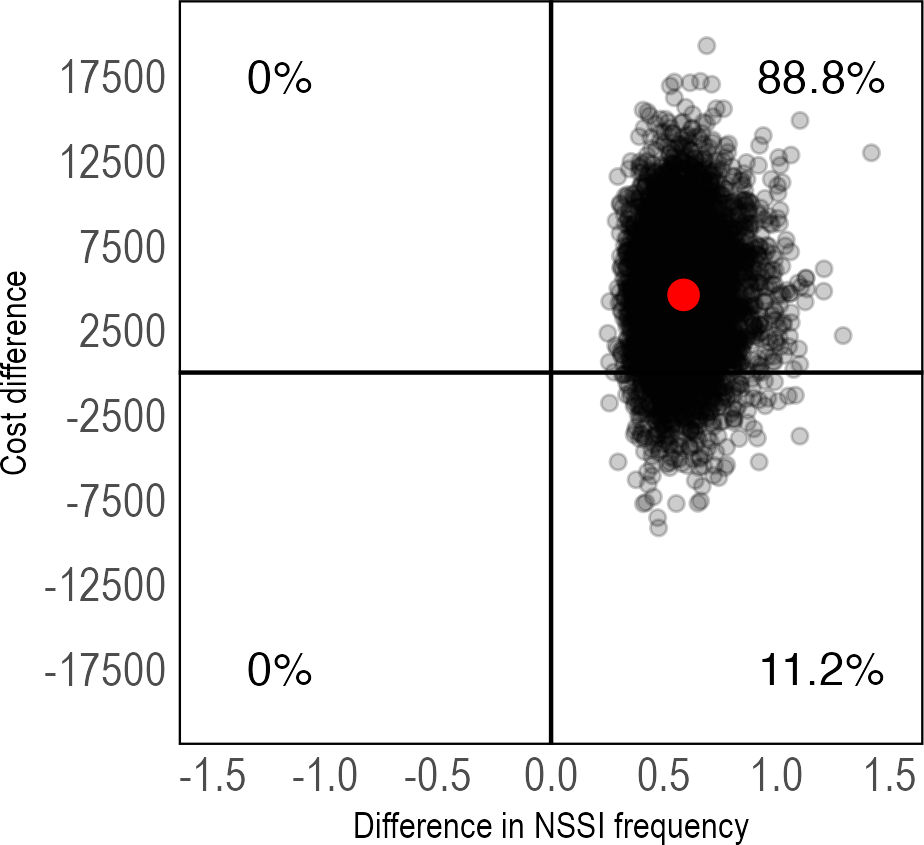
Pretreatment to 3-months posttreatment cost-effectiveness plane of IERITA plus TAU compared to TAU only, NSSI frequency. The x-axis displays between-group differences in outcomes, with positive values indicating more improvement in the IERITA plus TAU group compared to TAU only. Differences in costs, shown on the y-axis, are from a societal perspective, with positive values indicating increased costs in the IERITA plus TAU group compared to TAU only. IERITA: internet-delivered emotion regulation individual therapy for adolescents; NSSI: nonsuicidal self-injury; TAU: treatment as usual.

To estimate the cost-effectiveness of IERITA, differences in costs were divided by differences in the outcome variables of interest between IERITA plus TAU versus TAU only. The ICER for NSSI frequency on the masked clinician-assessed DSHI-Y was US $11,383 (95% CI US −$1611 to $29,299) per NSSI episode at 1-month posttreatment, and US $7814 (95% CI US −$5055 to $23,248) at 3-months posttreatment. A majority of ICERs from bootstrapped samples were located in the northeast quadrant at both 1-month posttreatment (Figure 2) and 3-months posttreatment (Figure 3), indicating higher costs and improved outcomes in the IERITA group. CEACs are included in Multimedia Appendix 1.

**Figure 4.**
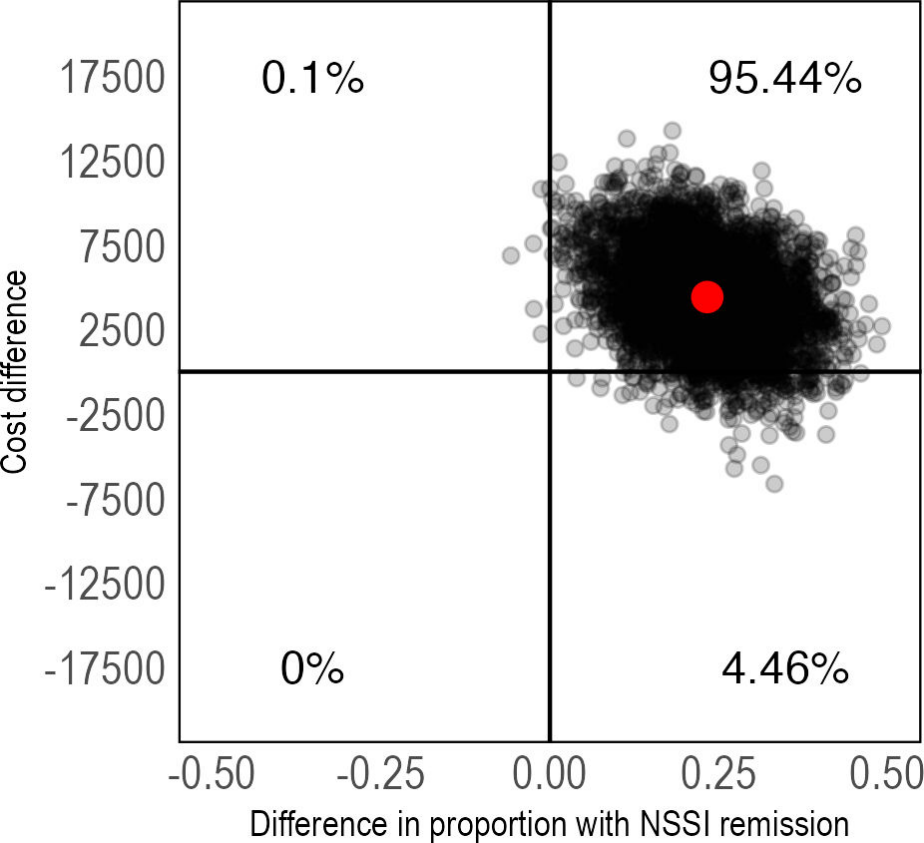
Pretreatment to 1-month posttreatment cost-effectiveness plane of IERITA plus TAU compared to TAU only, NSSI remission. The x-axis displays between-group differences in outcomes, with positive values indicating more improvement in the IERITA plus TAU group compared to TAU only. Differences in costs, shown on the y-axis, are from a societal perspective, with positive values indicating increased costs in the IERITA plus TAU group compared to TAU only. IERITA: internet-delivered emotion regulation individual therapy for adolescents; NSSI: nonsuicidal self-injury; TAU: treatment as usual.

The ICER for the outcome NSSI remission was US $18,677 (95% CI US $–2271 to $92,098) per additional participant with NSSI remission at 1-month posttreatment and US $27,563 (95% CI US $–775,711 to $737,706) at 3-months posttreatment, again with a majority of ICERs in the northeast quadrant at both time points (Figures4  5); however, with more uncertainty around the estimates at 3-months posttreatment. These results can assist decision makers by comparing the costs of existing treatment options for treating NSSI or by evaluating the value of NSSI remission in the current setting. This assessment should consider available health care resources, the potential need for costly inpatient care if NSSI is left untreated, and other relevant factors that may differ between health care systems (eg, unit costs for health care services and patient preferences).

**Figure 5.**
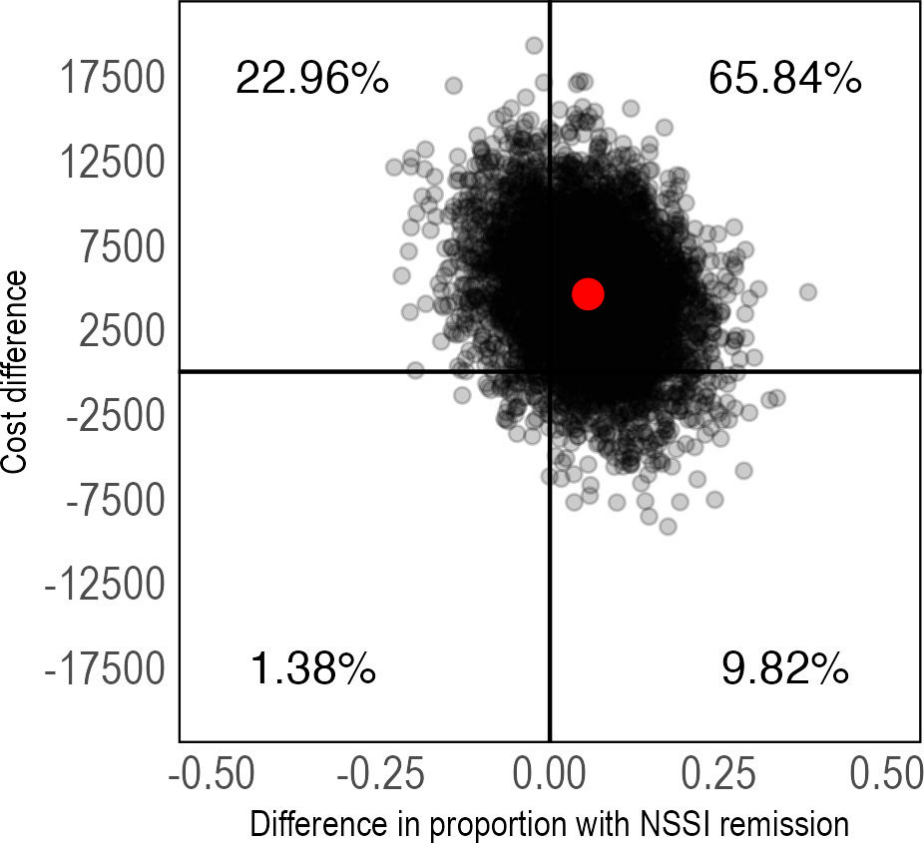
Pretreatment to 3-months posttreatment cost-effectiveness plane of IERITA plus TAU compared to TAU only, NSSI remission. The x-axis displays between-group differences in outcomes, with positive values indicating more improvement in the IERITA plus TAU group compared to TAU only. Differences in costs, shown on the y-axis, are from a societal perspective, with positive values indicating increased costs in the IERITA plus TAU group compared to TAU only. IERITA: internet-delivered emotion regulation individual therapy for adolescents; NSSI: nonsuicidal self-injury; TAU: treatment as usual.

**Figure 6.**
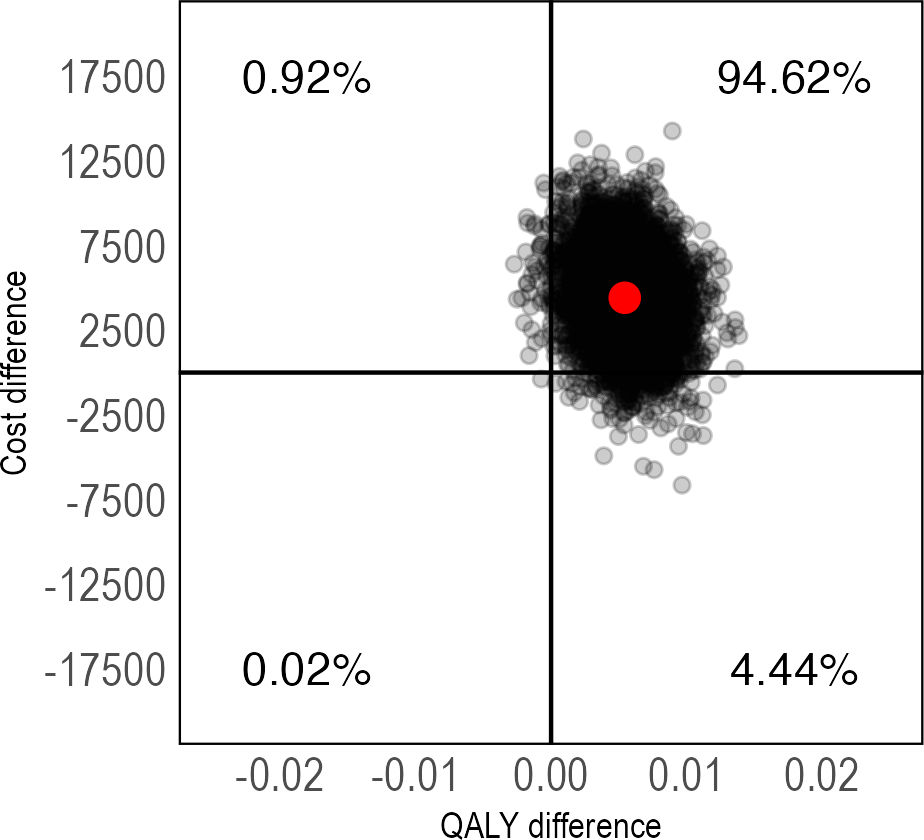
Pretreatment to 1-month posttreatment cost-utility of IERITA plus TAU compared to TAU only, QALYs. The x-axis displays between-group differences in outcomes, with positive values indicating more improvement in the IERITA plus TAU group compared to TAU only. Differences in costs, shown on the y-axis, are from a societal perspective, with positive values indicating increased costs in the IERITA plus TAU group compared to TAU only. IERITA: internet-delivered emotion regulation individual therapy for adolescents; NSSI: nonsuicidal self-injury; QALY: quality-adjusted life year; TAU: treatment as usual.

The cost-utility analyses showed that IERITA had an ICER of US $792,244 (95% CI US −$184,693 to $4,428,098) per QALY gained at 1-month posttreatment and US $471,800 (95% CI US −$428,322 to $4,272,413) at 3-months posttreatment. Again, the majority of ICERs were located in the northeast quadrant (Figures6  7). IERITA had a probability of being cost-effective of 8% at the Swedish reference value of US $84,000 at 1-month posttreatment [51]. Cost-effectiveness at 3-months posttreatment was 18% at a societal willingness-to-pay of US $84,000. Cost-effectiveness results on the self-rated DSHI-Y were similar (Multimedia Appendix 1).

**Figure 7.**
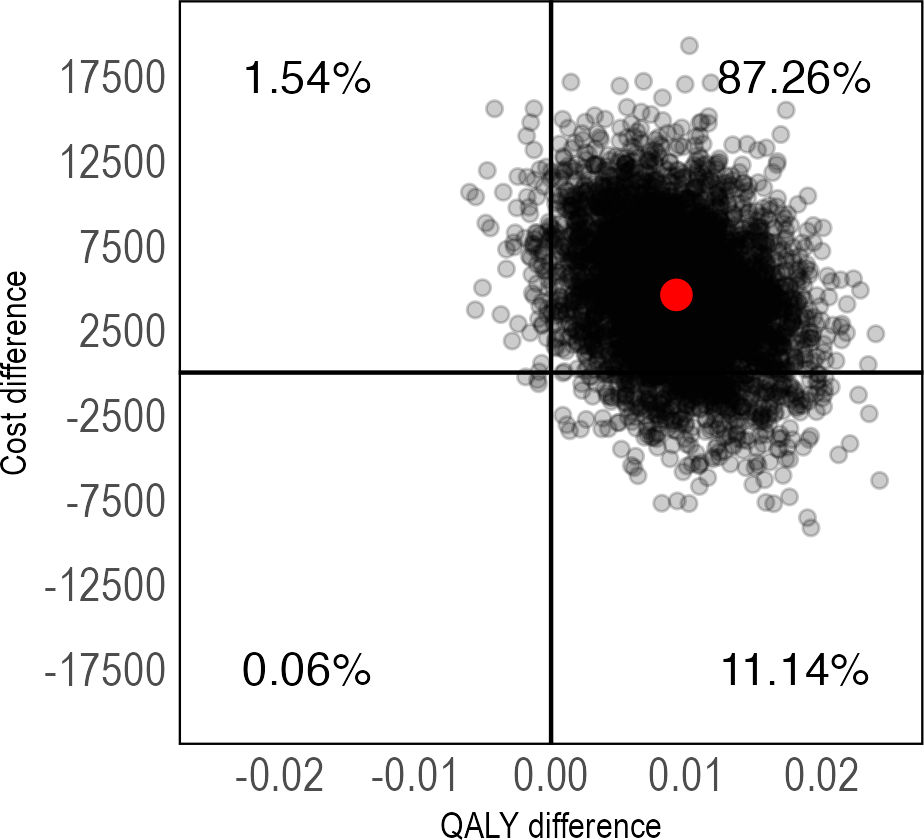
Pretreatment to 3-months posttreatment cost-utility of IERITA plus TAU compared to TAU only, QALYs. The x-axis displays between-group differences in outcomes, with positive values indicating more improvement in the IERITA plus TAU group compared to TAU only. Differences in costs, shown on the y-axis, are from a societal perspective, with positive values indicating increased costs in the IERITA plus TAU group compared to TAU only. IERITA: internet-delivered emotion regulation individual therapy for adolescents; NSSI: nonsuicidal self-injury; QALYs: quality-adjusted life years; TAU: treatment as usual.

#### The Health Care Perspective

From the health care perspective, which includes costs of health care visits and medications (but excludes nonmedical or indirect costs), the ICER on the masked clinician-assessed DSHI-Y was US $9534 (95% CI US $4895-$16,836) per NSSI episode at 1-month posttreatment and US $5952 (95% CI US $2328-$11,303) at 3-months posttreatment. The ICER of NSSI remission was US $15,502 (95% CI US $7203-$51,543) per additional participant with NSSI remission at 1-month posttreatment and US $29,972 (95% CI US −$488,780 to $467,794) at 3-months posttreatment. The ICER for the cost-utility analysis was US $660,793 (95% CI US $265,242 to $3,072,206) per QALY gained at 1-month posttreatment and US $361,041 (95% CI US $78,104 to $2,162,667) at 3-months posttreatment. The probability of IERITA being cost-effective was 1% at 1-month posttreatment and 3% at 3-months posttreatment. Cost-effectiveness planes and CEACs from the health care perspective are included in Multimedia Appendix 1.

#### Sensitivity Analyses

Cost-effectiveness and cost-utility estimates were similar when using observed data only (ie, no missing data imputation). At 1-month posttreatment, the ICER on the DSHI-Y was US $3558/1.04=US $3434, the ICER of NSSI remission was US $3558/0.23=US $15,548, and the cost per QALY was US $3558/0.005=US $660,968. At 3-months posttreatment, the ICER on the DSHI-Y was US $4429/1.04=US $7872, the ICER of NSSI remission was US $4429/0.08=US $58,111, and the cost per QALY was US $4429/0.009=US $488,623 (see Multimedia Appendix 1 for cost-effectiveness planes and CEACs).

As school-related costs accounted for the single largest proportion of costs, we calculated societal costs excluding this category. This resulted in similar between-group differences in costs at both 1-month posttreatment (US $4206, 95% CI US $1720-$6442) and 3-month follow-up (US $4548, 95% CI US $1103-$7767). Removing this category from the analyses, therefore, did not impact the overall conclusions.

To evaluate whether the participating youth received additional counseling outside the purview of their parents, youth- and parent-reported counseling use was compared at 1-month and 3-months posttreatment. There was agreement in 79%‐81% of cases. Disagreement was due to additional service use reported by either the youth (8% at 1-month posttreatment, 11% at 3-months posttreatment) or their parents (11% at both time points), and there was no evidence of systematic under or overreporting by parents (Multimedia Appendix 1).

## Discussion

### Principal Findings

The purpose of this study was to evaluate the cost-effectiveness and cost-utility of adding therapist-guided internet-delivered emotion regulation treatment (IERITA) to TAU for adolescents with NSSI, compared to TAU only. Our results showed that adding IERITA to TAU led to statistically significant reductions in NSSI, a higher proportion of participants with NSSI remission, and more QALYs, at 1-month posttreatment, at additional costs from health care and societal perspectives. The CIs of the ICERs of both NSSI frequency and NSSI remission were wide, ranging from lower costs and greater effects to higher costs and greater effects for IERITA+TAU relative to TAU. IERITA had an 8% probability of being cost-effective at a societal willingness-to-pay threshold of US $84,000 for one QALY at 1-month posttreatment, which increased to 18% at the 3-months posttreatment. This study provides an estimate of the cost of delivering IERITA delivered adjunctive to TAU, which is important for future implementation efforts.

The costs of providing IERITA adjunctive to TAU (US $3552) are higher than other internet-delivered treatments for other disorders in youth, for example, obsessive-compulsive disorder (US $2140 [53]), but substantially lower compared to high-intensity treatments such as DBT-A, which has an estimated treatment cost of €15,850 (US $15,052) per patient [17]. In the same study, DBT-A was found to be cost-saving over a 71-week period compared to eTAU, a difference that was largely driven by two long episodes of inpatient treatment in the eTAU group during the follow-up period [17]. Similarly, a brief cognitive behavioral therapy intervention for adults with self-harm was found to save £838 (US $680) in health care visits and sick-leave costs over 12 months compared to TAU [54]. In contrast, in this study, IERITA was associated with increased costs compared to TAU. However, direct comparisons between studies are challenging due to differences in evaluated costs (eg, only self-harm-related health care visits were measured in the DBT-A study versus all health care visits in this study), assessed health outcomes (eg, outcome was total number of self-harm episodes during follow-up in the DBT-A study vs weekly NSSI frequency in this study), follow-up duration, and health care context. The cost-effectiveness of IERITA may be improved if the treatment can be delivered at a lower cost (eg, automating parts of the therapists’ work such as responding to frequently asked questions), if IERITA is associated with lowered health care resource use through fewer parallel care contacts (discussed below), and longer follow-up periods to assess the full effect of IERITA on QALYs and costs (eg, whether IERITA protects against further deterioration of NSSI and thus need for more costly interventions in the future).

### Strengths and Limitations

This study has several strengths. First, the participants were recruited at three different child and adolescent psychiatric units, and the majority were referred by clinicians. The included participants are therefore more likely to be representative of the population seen in general psychiatric care than what would have been the case with a primarily self-referred sample. Second, the comparator was TAU within this psychiatric context, providing a realistic estimate of the costs and effects associated with the care typically provided to adolescents with NSSI in Sweden. Third, the platform carefully logged therapist activity, and it is therefore likely that the estimates of the treatment costs associated with the delivery of IERITA are accurate.

Nonetheless, some limitations deserve mention. First, most of the data collection relied on self-reports by parents, as well as therapists (time spent on phone contacts). This may have introduced bias into the estimates, which may differ from official records. However, the patient report is the preferred method to collect data on resource use and lost productivity [55], and the therapists followed guidelines for how to record the time spent on phone contacts, which was identical across both groups. Second, the 12-week recall period increases the risk of recall bias compared to shorter time frames. However, recall periods up to 6 months have been found to converge closely with weekly cost diary estimates [56]. Third, although QALY estimates were derived from KidScreen-10 scores using a validated algorithm [35], there is a risk that this indirect estimate reduced the precision in QALY estimates, particularly since the validation was made in another country (Australia) and on a general population sample of adolescents. Fourth, the costs and effects were limited to 3 months posttreatment, and we were not able to evaluate whether the treatment was associated with cost savings in the longer term, which has been found for DBT-A in adolescents and brief CBT in adults [17,54]. There are opportunities to address these limitations in future studies by incorporating registry-based estimates of resource use to evaluate the validity of self-reported costs in this context, using a questionnaire such as the CHU9D, where QALY estimates are directly available, and measuring resource use over a longer time period to evaluate long-term costs and effects of the intervention.

This is the first randomized clinical trial of internet-delivered treatment for adolescents with NSSI and NSSID, and one of the few trials demonstrating treatment effects in favor of the experimental intervention, regardless of treatment modality. The results, particularly the robust estimates of intervention cost, from this study can inform decision makers interested in implementing treatments for youth NSSI in clinical practice. For example, our findings show that providing IERITA as an adjunctive treatment to TAU is associated with statistically significant reductions in NSSI, a higher proportion of participants with no NSSI, and more QALYs at 1-month posttreatment, at an intervention cost of US $3552. Thus, for a relatively low cost compared to existing treatment options, this scalable and transportable treatment can provide public health benefits for a vulnerable population, otherwise at great risk for adverse outcomes. Because the treatment was considered an experimental intervention when the clinical trial took place, participants were recommended to continue or even establish other types of care contacts while also receiving IERITA. Future evaluations may consider using IERITA as a stand-alone first-line treatment with a reduced need for other ongoing care contacts, thus potentially offsetting the intervention costs of IERITA with lower health care costs from other sources. As discussed above, whether IERITA leads to similar long-term reductions in health care costs seen in other treatments is currently unknown. Prior to implementation, however, differences between this study and the specific context in which the intervention is to be implemented need to be considered. For instance, this study assessed resource use in a Swedish context, where prices for medications and health care visits are subsidized at the national level and are thus not paid in full by patients themselves. Differences in health care systems in other countries may thus affect changes in patient behavior during treatment (eg, rejecting other types of interventions while receiving IERITA in order to minimize costs). Furthermore, this study did not account for costs related to the development of the treatment protocol, server expenses, or licensing fees, which should be considered prior to implementation in other health care settings.

### Conclusions

Existing treatment options for adolescents with NSSI are often resource-intensive and costly. Here, we show that an internet-delivered emotion regulation therapy (IERITA) delivered adjunctive to TAU led to improvements in NSSI frequency, remission, and QALYs at additional costs, compared to TAU only. This study provides an estimate of the additional cost of delivering IERITA compared to TAU. However, the full effects of an intervention on QALYs and societal costs are likely seen over longer time periods (eg, whether the treatment is associated with a reduced need for other costly interventions or can improve school attendance), and studies of IERITA including longer follow-up periods are therefore needed.

## Supplementary material

10.2196/74303 Multimedia Appendix 1 Additional details on cost sources, detailed TIC-P results, and sensitivity analyses.

## Abbreviations

CEAC: cost-effectiveness acceptability curve
CHEERS: Consolidated Health Economic Evaluation Reporting Standards
CHU9D: Child Health Utility 9D
DBT-A: dialectical behavior therapy for adolescents
DSHI-Y: Deliberate Self-Harm Inventory-Youth version
eTAU: enhanced treatment as usual
ICER: incremental cost-effectiveness ratio
IERITA: internet-delivered emotion regulation individual therapy for adolescents
IRR: incidence rate ratio
NSSI: nonsuicidal self-injury
NSSID: nonsuicidal self-injury disorder
QALY: quality-adjusted life-year
TAU: treatment as usual
TIC-P: Trimbos-iMTA questionnaire for costs associated with psychiatric illness

## References

[1] Ribeiro JD, Franklin JC, Fox KR (2016). Self-injurious thoughts and behaviors as risk factors for future suicide ideation, attempts, and death: a meta-analysis of longitudinal studies. Psychol Med.

[2] Bjureberg J, Ohlis A, Ljótsson B (2019). Adolescent self-harm with and without suicidality: cross-sectional and longitudinal analyses of a Swedish regional register. J Child Psychol Psychiatry.

[3] American Psychiatric Association, DSM-5 Task Force (2013). Diagnostic and Statistical Manual of Mental Disorders: DSM-5.

[4] Zetterqvist M, Lundh LG, Dahlström O, Svedin CG (2013). Prevalence and function of non-suicidal self-injury (NSSI) in a community sample of adolescents, using suggested DSM-5 criteria for a potential NSSI disorder. J Abnorm Child Psychol.

[5] Zetterqvist M (2015). The DSM-5 diagnosis of nonsuicidal self-injury disorder: a review of the empirical literature. Child Adolesc Psychiatry Ment Health.

[6] Mars B, Heron J, Crane C (2014). Clinical and social outcomes of adolescent self harm: population based birth cohort study. BMJ.

[7] Dyvesether SM, Hastrup LH, Hawton K, Nordentoft M, Erlangsen A (2022). Direct costs of hospital care of self-harm: a national register-based cohort study. Acta Psychiatr Scand.

[8] Sinclair JMA, Gray A, Rivero-Arias O, Saunders KEA, Hawton K (2011). Healthcare and social services resource use and costs of self-harm patients. Soc Psychiatry Psychiatr Epidemiol.

[9] Bjureberg J, Kuja-Halkola R, Ohlis A (2022). Adverse clinical outcomes among youths with nonsuicidal self-injury and suicide attempts: a longitudinal cohort study. J Child Psychol Psychiatry.

[10] Large MM, Chung DT, Davidson M, Weiser M, Ryan CJ (2017). In-patient suicide: selection of people at risk, failure of protection and the possibility of causation. BJPsych Open.

[11] Epstein S, Roberts E, Sedgwick R (2020). School absenteeism as a risk factor for self-harm and suicidal ideation in children and adolescents: a systematic review and meta-analysis. Eur Child Adolesc Psychiatry.

[12] Pitkänen J, Remes H, Aaltonen M, Martikainen P (2023). Moderating role of sociodemographic factors in parental psychiatric treatment before and after offspring severe self-harm. J Affect Disord.

[13] Miller AL, Rathus JH, Linehan M (2007). Dialectical Behavior Therapy with Suicidal Adolescents.

[14] Mehlum L, Tørmoen AJ, Ramberg M (2014). Dialectical behavior therapy for adolescents with repeated suicidal and self-harming behavior: a randomized trial. J Am Acad Child Adolesc Psychiatry.

[15] McCauley E, Berk MS, Asarnow JR (2018). Efficacy of dialectical behavior therapy for adolescents at high risk for suicide: a randomized clinical trial. JAMA Psychiatry.

[16] Asarnow JR, Ougrin D (2019). Editorial: Suicide and self‐harm: advancing from science to preventing deaths. Child Psychology Psychiatry.

[17] Haga E, Aas E, Grøholt B, Tørmoen AJ, Mehlum L (2018). Cost-effectiveness of dialectical behaviour therapy vs. enhanced usual care in the treatment of adolescents with self-harm. Child Adolesc Psychiatry Ment Health.

[18] Bjureberg J, Sahlin H, Hedman-Lagerlöf E (2018). Extending research on Emotion Regulation Individual Therapy for Adolescents (ERITA) with nonsuicidal self-injury disorder: open pilot trial and mediation analysis of a novel online version. BMC Psychiatry.

[19] Morthorst B, Rubæk L, Lindschou J (2021). An internet-based emotion regulation intervention versus no intervention for nonsuicidal self-injury in adolescents: study protocol for a feasibility trial. Pilot Feasibility Stud.

[20] Simonsson O, Engberg H, Bjureberg J (2021). Experiences of an online treatment for adolescents with nonsuicidal self-injury and their caregivers: qualitative study. JMIR Form Res.

[21] Bjureberg J, Ojala O, Hesser H (2023). Effect of internet-delivered emotion regulation individual therapy for adolescents with nonsuicidal self-injury disorder: a randomized clinical trial. JAMA Netw Open.

[22] Husereau D, Drummond M, Augustovski F (2022). Consolidated Health Economic Evaluation Reporting Standards 2022 (CHEERS 2022) statement: updated reporting guidance for health economic evaluations. MDM Policy Pract.

[23] Bjureberg J, Sahlin H, Hellner C (2017). Emotion regulation individual therapy for adolescents with nonsuicidal self-injury disorder: a feasibility study. BMC Psychiatry.

[24] Hakkaart-van Roijen L, Van Straten A, Donker M, Tiemens B (2002). Manual Trimbos/iMTA questionnaire for costs associated with psychiatric illness (TiC-P).

[25] (2024). Prices of medical products and medications. https://www.tlv.se/beslut/sok-priser-och-beslut-i-databasen.html.

[26] Neumann P, Sanders GD, Russell LB, Siegel JE, Ganiats TG (2017). Cost-Effectiveness in Health and Medicine.

[27] (2024). Consumer price index (CPI). Statistics Sweden.

[28] (2024). Labour cost index (LCI). Statistics Sweden.

[29] (2024). Salary structures, whole economy. https://www.statistikdatabasen.scb.se/pxweb/en/ssd/START__AM__AM0110__AM0110A/LonYrkeUtbildning4A/.

[30] (2024). Purchasing power parities (PPP). OECD.

[31] Gratz KL, Latzman RD, Young J (2012). Deliberate self-harm among underserved adolescents: the moderating roles of gender, race, and school-level and association with borderline personality features. Personality Disorders: Theory, Research, and Treatment.

[32] Gratz KL (2001). Measurement of deliberate self-harm: preliminary data on the deliberate self-harm inventory. J Psychopathol Behav Assess.

[33] Ravens-Sieberer U, Wille N, Badia X (2010). Feasibility, reliability, and validity of the EQ-5D-Y: results from a multinational study. Qual Life Res.

[34] Erhart M, Ottova V, Gaspar T (2009). Measuring mental health and well-being of school-children in 15 European countries using the KIDSCREEN-10 Index. Int J Public Health.

[35] Chen G, Stevens K, Rowen D, Ratcliffe J (2014). From KIDSCREEN-10 to CHU9D: creating a unique mapping algorithm for application in economic evaluation. Health Qual Life Outcomes.

[36] Stevens K (2011). Assessing the performance of a new generic measure of health-related quality of life for children and refining it for use in health state valuation. Appl Health Econ Health Policy.

[37] Stevens K (2012). Valuation of the Child Health Utility 9D index. Pharmacoeconomics.

[38] Matthews JN, Altman DG, Campbell MJ, Royston P (1990). Analysis of serial measurements in medical research. BMJ.

[39] Faria R, Gomes M, Epstein D, White IR (2014). A guide to handling missing data in cost-effectiveness analysis conducted within randomised controlled trials. Pharmacoeconomics.

[40] van Buuren S (2007). Multiple imputation of discrete and continuous data by fully conditional specification. Stat Methods Med Res.

[41] El Alili M, van Dongen JM, Esser JL, Heymans MW, van Tulder MW, Bosmans JE (2022). A scoping review of statistical methods for trial-based economic evaluations: the current state of play. Health Econ.

[42] Brand J, van Buuren S, le Cessie S, van den Hout W (2019). Combining multiple imputation and bootstrap in the analysis of cost-effectiveness trial data. Stat Med.

[43] Briggs A, Nixon R, Dixon S, Thompson S (2005). Parametric modelling of cost data: some simulation evidence. Health Econ.

[44] Ben ÂJ, van Dongen JM, El Alili M, Esser JL, Broulíková HM, Bosmans JE (2023). Conducting trial-based economic evaluations using R: a tutorial. Pharmacoeconomics.

[45] Manca A, Hawkins N, Sculpher MJ (2005). Estimating mean QALYs in trial-based cost-effectiveness analysis: the importance of controlling for baseline utility. Health Econ.

[46] Drummond MF, Sculpher MJ, Claxton K, Stoddart GL, Torrance GW (2015). Methods for the Economic Evaluation of Health Care Programmes.

[47] Efron B, Tibshirani R (1986). Bootstrap methods for standard errors, confidence intervals, and other measures of statistical accuracy. Statist Sci.

[48] Black WC (1990). The CE plane: a graphic representation of cost-effectiveness. Med Decis Making.

[49] Fenwick E, Claxton K, Sculpher M (2001). Representing uncertainty: the role of cost-effectiveness acceptability curves. Health Econ.

[50] Shiroiwa T, Sung YK, Fukuda T, Lang HC, Bae SC, Tsutani K (2010). International survey on willingness-to-pay (WTP) for one additional QALY gained: what is the threshold of cost effectiveness?. Health Econ.

[51] Nilsson FOL, Svensson M, Arnberg K (2014). Reimbursement decisions for pharmaceuticals in Sweden: the impact of cost-effectiveness and disease severity. Value Health.

[52] Bjureberg J, Ojala O (2024). Cost-effectiveness of internet-delivered emotion regulation individual therapy for adolescents with nonsuicidal self-injury. PsyArXiv.

[53] Aspvall K, Sampaio F, Lenhard F (2021). Cost-effectiveness of internet-delivered vs in-person cognitive behavioral therapy for children and adolescents with obsessive-compulsive disorder. JAMA Netw Open.

[54] Byford S, Knapp M, Greenshields J (2003). Cost-effectiveness of brief cognitive behaviour therapy versus treatment as usual in recurrent deliberate self-harm: a decision-making approach. Psychol Med.

[55] van Lier LI, Bosmans JE, van Hout HPJ (2018). Consensus-based cross-European recommendations for the identification, measurement and valuation of costs in health economic evaluations: a European Delphi study. Eur J Health Econ.

[56] van den Brink M, van den Hout WB, Stiggelbout AM, Putter H, van de Velde CJH, Kievit J (2005). Self-reports of health-care utilization: diary or questionnaire?. Int J Technol Assess Health Care.

