# Supplementary material for: Cost-Effectiveness of Internet-Delivered Emotion Regulation Therapy for Adolescents With Nonsuicidal Self-Injury: Within-Trial Analysis of a Randomized Controlled Trial
Source: JMIR Ment Health. 2025 Aug 27;12:e74303. doi: 10.2196/74303 (PMC12382421; doi:10.2196/74303)
Supplement: Multimedia Appendix 1 [file mental-v12-e74303-s001.docx]

Cost-effectiveness of Internet-Delivered Emotion Regulation Therapy for Adolescents With Nonsuicidal Self-Injury

Table of contents

[eMethods 1](#_Toc171954459)

[Resource use and costs, continued 1](#_Toc171954460)

[eResults 3](#_Toc171954461)

[Detailed TIC-P results 3](#_Toc171954462)

[Parent participation during treatment 4](#_Toc171954463)

[Self-rated NSSI results 4](#_Toc171954464)

[Cost-effectiveness acceptability curves 5](#_Toc171954465)

[Healthcare perspective 7](#_Toc171954466)

[Observed data, societal perspective 9](#_Toc171954467)

[Healthcare perspective cost-effectiveness figures 10](#_Toc171954468)

[Cost-effectiveness and cost-utility using observed data 13](#_Toc171954469)

[Agreement between parent and youth reports of other counselling 15](#_Toc171954470)

[References 15](#_Toc171954471)

# eMethods

## Resource use and costs, continued

Table S1 shows the tariffs and unit costs, along with sources, for the costs included in the cost-effectiveness analysis. The costs are presented in 2022 US dollars.

| Table S1. Unit Costs and Sources, in 2022 USD | | |
| --- | --- | --- |
| **Resource item** | **Unit cost, $^a^** | **Source, comment** |
| *Healthcare resources (per visit)* | | |
| General practitioner | 286 | Sweden’s municipalities and regions |
| Specialist physician^b^ | 1068 | Sweden’s municipalities and regions |
| Psychologist | 487 | Sweden’s municipalities and regions |
| Nurse | 99 | Sweden’s municipalities and regions |
| Other professions^c^ | 487 | Sweden’s municipalities and regions |
| Inpatient psychiatric care | 1574 + 1010 per day of care | Sweden’s municipalities and regions |
| *Medication* | | |
| Medicines | Individual product prices | The Dental and Pharmaceutical Benefits Agency of Sweden |
| Dietary supplements | Individual product prices | Market price from Swedish pharmacy |
| *Support and assistance (per hour)* | | |
| Specialist teacher | 46 | Statistics Sweden |
| Study help | 54 | Own estimate^d^ |
| Support from family and friends | 25 | Estimated as cost of leisure time, posttax wage/h in Sweden |
| Personal assistant | 20 | Sweden’s municipalities and regions |
| Support family | 24 | Sweden’s municipalities and regions |
| *Productivity losses* | | |
| Cost per child/d at school^e^ | 88 | Own estimated based on Swedish National Agency for Education |
| Average wage/hour in Sweden^f^ | 41 | Statistics Sweden |
| Cost of leisure time/h | 25 | Posttax wage/h in Sweden (Neumann et al., 2017) |
| *Intervention cost* | | |
| Psychologist/h^g^ | 598 | Sweden’s municipalities and regions |
| ^a^All costs uprated to 2022 US dollars.  ^b^Visit to specialist physician within child and adolescent psychiatry  ^c^Physiotherapist, dietician, counsellor, or speech therapist  ^d^Market prices from a popular study help provider in Sweden  ^e^Average annual cost per pupil and year, divided by the number of school days per year.  ^f^Includes social fees of 43.3%. Used to estimate productivity losses due to absence from work.  ^g^Cost of psychologist working in child and adolescent outpatient psychiatric units | | |

# eResults

## Detailed TIC-P results

In the tables below, costs from various sources in the TIC-P are shown separately. Costs for providing therapy (i.e., therapist time) are not included. All costs are in 2022 US dollars and aggregated over the trial period.

| Table S2: TIC-P costs at baseline. Mean (SD), median values.   \| TIC-P category \| TAU only \| IERITA plus TAU \| \| --- \| --- \| --- \| \| Health-care visits \| $4172 ($3920), $3006 \| $4443 ($3921), $3072 \| \| Social support and assistance \| $1184 ($2004), $440 \| $958 ($1552), $407 \| \| Medications \| $120 ($616), $2 \| $45 ($118), $6 \| \| Supplements \| $5 ($11), $0 \| $3 ($9), $0 \| \| Parent unemployment \| $1754 ($6619), $0 \| $1713 ($7280), $0 \| \| Parental absenteeism \| $640 ($1536), $0 \| $702 ($1717), $0 \| \| School absenteeism and presenteeism \| $13363 ($13373), $9151 \| $16347 ($17824), $8895 \| |
| --- | --- | --- | --- | --- | --- | --- | --- | --- | --- | --- | --- | --- | --- | --- | --- | --- | --- | --- | --- | --- | --- | --- | --- | --- |

| Table S3: TIC-P costs at 1-month post-treatment. Mean (SD), median values.   \| TIC-P category \| TAU only \| IERITA plus TAU \| \| --- \| --- \| --- \| \| Health-care visits \| $3590 ($4732), $2115 \| $4232 ($4507), $2991 \| \| Social support and assistance \| $884 ($1692), $204 \| $981 ($1591), $244 \| \| Medications \| $134 ($637), $8 \| $49 ($117), $3 \| \| Supplements \| $7 ($16), $0 \| $3 ($7), $0 \| \| Parent unemployment \| $811 ($4028), $0 \| $1561 ($6391), $0 \| \| Parental absenteeism \| $1063 ($3217), $0 \| $692 ($1617), $0 \| \| School absenteeism and presenteeism \| $11584 ($14337), $7489 \| $13188 ($15854), $7758 \| |
| --- | --- | --- | --- | --- | --- | --- | --- | --- | --- | --- | --- | --- | --- | --- | --- | --- | --- | --- | --- | --- | --- | --- | --- | --- |

| Table S4: TIC-P costs at 3-months post-treatment. Mean (SD), median values.   \| TIC-P category \| TAU only \| IERITA plus TAU \| \| --- \| --- \| --- \| \| Health-care visits \| $5731 ($6419), $3659 \| $6339 ($6266), $4357 \| \| Social support and assistance \| $1484 ($2995), $462 \| $1586 ($2790), $432 \| \| Medications \| $222 ($1074), $14 \| $91 ($202), $13 \| \| Supplements \| $11 ($24), $0 \| $5 ($11), $0 \| \| Parent unemployment \| $1032 ($4373), $0 \| $2558 ($9740), $0 \| \| Parental absenteeism \| $1569 ($4213), $0 \| $1162 ($2417), $0 \| \| School absenteeism and presenteeism \| $17976 ($21118), $10533 \| $20523 ($23940), $9978 \| |
| --- | --- | --- | --- | --- | --- | --- | --- | --- | --- | --- | --- | --- | --- | --- | --- | --- | --- | --- | --- | --- | --- | --- | --- | --- |

## Parent participation during treatment

The costs associated with parents supporting their child during the study period are shown in Table S5. Note that these costs are not aggregated but shown for each time-point separately. All costs are in 2022 US dollars.

| Table S5: Costs associated with parent involvement. Observed values for each time-point separately. Mean (SD), median values.   \| time \| TAU only \| IERITA plus TAU \| \| --- \| --- \| --- \| \| Baseline \| $1477 ($2781), $429 \| $1214 ($2134), $501 \| \| 1-month post-treatment \| $1044 ($2196), $286 \| $1119 ($1758), $343 \| \| 3-months post-treatment \| $970 ($2728), $143 \| $797 ($2286), $100 \| |
| --- | --- | --- | --- | --- | --- | --- | --- | --- | --- | --- | --- | --- |

## Self-rated NSSI results

At 1-month post-treatment, there was an estimated lower rate of NSSI in the IERITA plus TAU group (0.85) compared to TAU only (2.69), with an estimate of 1.85 fewer episodes per week on the DSHI-Y. Cost-effectiveness from a societal perspective on the self-rated DSHI-Y from pre-treatment to 1-month post-treatment was $4458 / 1.85 = $2409, see [Figure S1](#fig-dshiy-self-post). When including only healthcare costs, the cost-effectiveness was $3663 / 1.85 = $1980. The proportion of missing values on the self-rated DSHI-Y was 0% at baseline, 3-25% at weekly assessments, and 19 (11%) missing responses at 1-month post-treatment.

|   Figure S1: Pre-treatment to 1-month post-treatment cost-effectiveness of IERITA plus TAU compared to TAU only. Between-group difference on self-rated DSHI-Y, positive values indicate more improvement in the IERITA plus TAU group. Differences in costs from a societal perspective are shown on the y-axis, with positive values indicating increased costs in the IERITA plus TAU group compared to TAU only. |
| --- |

## Cost-effectiveness acceptability curves

Cost-effectiveness acceptability curves when using costs from a societal perspective are shown in [Figures S2](#fig-wtp-post) and [S3](#fig-wtp-followup) for NSSI frequency, NSSI remission, and QALYs. The curves show the probability of IERITA plus TAU being cost-effective compared to TAU only at different willingness-to-pay thresholds, based on the bootstrapped estimates.

|   Figure S2: Pre-treatment to 1-month post-treatment cost-effectiveness acceptability curves for NSSI frequency, NSSI remission and QALYs. IERITA plus TAU compared to TAU only. Costs are from a societal perspective. |
| --- |
|   Figure S3: Pre-treatment to 3-months post-treatment cost-effectiveness acceptability curves for NSSI frequency, NSSI remission and QALYs. IERITA plus TAU compared to TAU only. Costs are from a societal perspective. |

### Healthcare perspective

|   Figure S4: Pre-treatment to 1-month post-treatment cost-effectiveness acceptability curves for NSSI frequency, NSSI remission and QALYs. IERITA plus TAU compared to TAU only. Costs are from a healthcare perspective. |
| --- |
|   Figure S5: Pre-treatment to 3-months post-treatment cost-effectiveness acceptability curves for NSSI frequency, NSSI remission and QALYs. IERITA plus TAU compared to TAU only. Costs are from a healthcare perspective. |

### Observed data, societal perspective

|   Figure S6: Pre-treatment to 1-month post-treatment cost-effectiveness acceptability curves for NSSI frequency, NSSI remission and QALYs. IERITA plus TAU compared to TAU only. Costs are from a societal perspective. Bootstrap estimates from observed data with no missing data imputation. |
| --- |
|   Figure S7: Pre-treatment to 3-months post-treatment cost-effectiveness acceptability curves for NSSI frequency, NSSI remission and QALYs. IERITA plus TAU compared to TAU only. Costs are from a societal perspective. Bootstrap estimates from observed data with no missing data imputation. |

## Healthcare perspective cost-effectiveness figures

In [Figure S8](#fig-hc-post), the cost-effectiveness planes of NSSI frequency, NSSI remission, and QALYs at 1-month post-treatment from a healthcare perspective are shown. Similarly in [Figure S9](#fig-hc-followup), the same outcomes are shown at 3-months post-treatment using costs from a healthcare perspective.

|   Figure S8: Pre-treatment to 1-month post-treatment cost-effectiveness and cost-utility of IERITA plus TAU compared to TAU only. Cost-effectiveness planes for NSSI frequency (panel A), NSSI remission (panel B), and QALYs (panel C). The x-axis displays between-group differences in outcomes, with positive values indicating more improvement in the IERITA plus TAU group compared to TAU only. Differences in costs, shown on the y-axis, are from a healthcare perspective, with positive values indicating increased costs in the IERITA plus TAU group compared to TAU only. Abbreviations: NSSI, non-suicidal self-injury; QALYs, Quality-adjusted life years. |
| --- |
|   Figure S9: Pre-treatment to 3-months post-treatment cost-effectiveness and cost-utility of IERITA plus TAU compared to TAU only. Cost-effectiveness planes for NSSI frequency (panel A), NSSI remission (panel B), and QALYs (panel C). The x-axis displays between-group differences in outcomes, with positive values indicating more improvement in the IERITA plus TAU group compared to TAU only. Differences in costs, shown on the y-axis, are from a healthcare perspective, with positive values indicating increased costs in the IERITA plus TAU group compared to TAU only. Abbreviations: NSSI, non-suicidal self-injury; QALYs, Quality-adjusted life years. |

## Cost-effectiveness and cost-utility using observed data

Cost-effectiveness planes for NSSI frequency, NSSI remission, and QALYs, from a societal perspective and using complete data only are shown at 1-month post-treatment in [Figure S10](#fig-missing-post) and 3-months post-treatment in [Figure S11](#fig-missing-followup).

|   Figure S10: Pre-treatment to 1-month post-treatment cost-effectiveness and cost-utility of IERITA plus TAU compared to TAU only. Cost-effectiveness planes for NSSI frequency (panel A), NSSI remission (panel B), and QALYs (panel C). The x-axis displays between-group differences in outcomes, with positive values indicating more improvement in the IERITA plus TAU group compared to TAU only. Differences in costs, shown on the y-axis, are from a societal perspective, with positive values indicating increased costs in the IERITA plus TAU group compared to TAU only. Abbreviations: NSSI, non-suicidal self-injury; QALYs, Quality-adjusted life years. |
| --- |
|   Figure S11: Pre-treatment to 3-months post-treatment cost-effectiveness and cost-utility of IERITA plus TAU compared to TAU only. Cost-effectiveness planes for NSSI frequency (panel A), NSSI remission (panel B), and QALYs (panel C). The x-axis displays between-group differences in outcomes, with positive values indicating more improvement in the IERITA plus TAU group compared to TAU only. Differences in costs, shown on the y-axis, are from a societal perspective, with positive values indicating increased costs in the IERITA plus TAU group compared to TAU only. Abbreviations: NSSI, non-suicidal self-injury; QALYs, Quality-adjusted life years. |

## Agreement between parent and youth reports of other counselling

Both participating youth and their parents responded to questions about other ongoing counselling during the study period. In order to evaluate whether teens received services without their parents’ knowledge, the responses were compared using the bias and prevalence adjusted kappa (PABAK; Byrt, Bishop, and Carlin (1993)). There was substantial agreement at 1-month post-treatment (kappa = 0.62, 95% CI 0.48 to 0.74) and fair agreement at 3-months post-treatment (kappa = 0.57, 95% CI 0.42 to 0.7), with no evidence of bias according to the McNemar test (*p* = 0.353 to .99). Specifically, at 1-month post-treatment there was agreement between parent and youth in 80.9% of cases, with parents reporting additional services in 11.2% of cases and the youth in 7.9% of cases. At 3-months post-treatment, there was agreement in 78.6% of cases and additional service use reported by parents or youth in 10.7% of cases each.

# References

Byrt, Ted, Janet Bishop, and John B. Carlin. 1993. “Bias, Prevalence and Kappa.” *Journal of Clinical Epidemiology* 46 (5): 423–29. <https://doi.org/10.1016/0895-4356(93)90018-V>.

Neumann, Peter, Gillian D. Sanders, Louise B. Russell, Joanna E. Siegel, and Theodore G. Ganiats, eds. 2017. *Cost-Effectiveness in Health and Medicine*. Second edition. New York, NY, United States of America: Oxford University Press.
